# Increasing Longevity

**Published:** 1894-06

**Authors:** 


### Increasing Longevity.

"The threescore and ten years allotted to man will yet be increased to twice that number," was the prediction made by Dr. Charles Hienkle. "Instead of the world growing weaker and wiser, as the old axiom puts it, it is growing wiser and stronger. The average length of life is steadily increasing. In the days of good Queen Bess women were considered passe at thirty, and few men distinguished themselves in state-craft, science or literature after passing five and sixty. Now a woman is in the heydey of her beauty at thirty, and the ripest fruits of genius are frequently plucked at threescore and ten. Gladstone, Bismarck and Blaine are fair examples of that green and fruitful old age so frequent in these days. Yet science is but in its infancy. As it progresses the waste of life and energy will be gradually curtailed.

"While the fountain of youth sought by that interesting old crank, Ponce de Leon, will probably never be found; while man will probably never discover the secret of remaining an ever young Apollo, nor woman that of being as attractive at sixty as at sixteen, I firmly believe that the meridian of life will yet be raised from thirty-five to seventy; that the day will come when a man will not be considered a back number when he has reached his 100th birthday."—*St. Louis Globe Democrat.*

Offensive Odor of the Breath may be overcome by the habitual use of Eucalyptus and Thymol Antiseptic. Add a teaspoonful to a tumblerful of water for a mouth-wash and gargle. For bad breath caused by gaseous eructations of indigestion, Eucalyptus and Thymol Antiseptic is an excellent remedy, in doses of twenty to thirty drops in a little water.

